# Competition for resources: complicated dynamics in the simple Tilman model

**DOI:** 10.1186/s40064-015-1246-6

**Published:** 2015-09-04

**Authors:** Joost H. J. van Opheusden, Lia Hemerik, Mieke van Opheusden, Wopke van der Werf

**Affiliations:** Biometris, Wageningen University, Droevendaalsesteeg 1, 6708PB Wageningen, The Netherlands; Crop Systems Analysis Group, Plant Sciences, Wageningen University, Droevendaalsesteeg 1, 6708PB Wageningen, The Netherlands

**Keywords:** Consumer, Resource competition, Mathematical model, Stability, Time scales

## Abstract

Graphical analysis and computer simulations have become the preferred tools to present Tilman’s model of resource competition to new generations of ecologists. To really understand the full dynamic behaviour, a more rigorous mathematical analysis is required. We show that just a basic stability analysis is insufficient to describe the relevant dynamics of this deceptively simple model. To investigate realistic invasion and succession processes, not only the stable state is relevant, but also the time scales at which the system moves away from the unstable situation. We argue that the relative stability of saddle points is more important for the actual observed transient dynamics in realistic systems than the predicted asymptotic behaviour towards the stable equilibria. For the mathematical analysis this implies that not only the signs, but also the magnitudes of the eigenvalues of the Jacobi matrix at the stationary points, the rates at which the system evolves, must be considered. We present the underlying mathematics of the Tilman model in a way that should be accessible to any ecologist with a basic mathematical background.

## Background

If the development of an ecosystem is driven by competition, why doesn’t a single species outcompetes all others and becomes the dominant, if not the only surviving one? Why don’t we always have food chains and food towers instead of food webs and food pyramids? Ecological models that describe the resource competition between different species help to understand biodiversity (Levin 2012). Models that show how and why in some situations several species can coexist, while in slightly different situations one may prevail over others, can explain observations of invasion and succession processes in real life environments. Even very simple models can exhibit such complicated behaviour, but unfortunately also the mathematics behind such simple models can become complicated (Edelstein-Keshet 2005). Graphical analysis tools and numerical simulations do enhance accessibility of these models, but cannot replace the in depth insight obtained from mathematical analysis. In fact, for a full understanding of the dynamics of the transition of a system from one situation to another, mathematical analysis is indispensable. Here, we summarize the elementary mathematics underlying a suite of simple models of resource competition (Tilman 1982), in which the dynamics of consumers and resources are explicitly represented. The original papers presenting the mathematical roots of the model require mathematical skills that most ecologists do not possess. Our aim is to make the presentation as accessible as possible. Thus, we keep mathematical sophistication to the minimum needed to explain how the ecology of the model derives from the mathematics.

In the model as introduced by Tilman there are consumers, plants or animals that are part of a given ecosystem, and resources, other plants or animals, but also water, minerals or light that the consumers need for their maintenance and growth. Initially the model was applied to competition between several species of algae (Tilman 1977), which may explain some specific choices made in the model, also when applied more generally. The state variables are the overall densities of consumers and resources within an ecosystem. The model describes the development of these densities in time. There is no direct interaction between the consumers, nor between the resources. Only in the interaction between the consumer and the various resources can there be a combined effect of the different resource densities. The dynamics of the densities is described mathematically by a system of coupled ordinary differential equations.

In “The Tilman model” we discuss the general form of the equations for multiple species competing for several resources, and critically discuss assumptions made in the model. In “Stability analysis of the Tilman model” the model is simplified, *c.f.* specified, to make it amenable for mathematical analysis, and we discuss what further biological implications are associated with the restricted model. It will turn out that to a large extent the model can be solved quite generically, without full specification of the interaction. We develop a stability analysis for a system of up to two different species competing for two resources, and recapitulate the earlier derived conditions for a stable coexistence. The conditions are mathematical relations between model parameters. Moreover, the analysis yields the relevant time scales at which the model operates, not only for the stable asymptotic state, but also for the unstable, possibly transient states.

To investigate the full dynamic behaviour of the models, which we do in “Numerical model calculations”, we will need to specify the interactions within the model further, and with those the stability results. For all models we investigate how a relatively unpopulated system develops towards the final state with one or two consumers coexisting with the resources. It turns out that indeed all stationary states are important for the overall dynamics, and also the rates, or inversely the time scales, at which the system moves away from the unstable states. Finally we review the biological assumptions made, and explain how we can understand the observed model behaviour in terms of its biological background, or, vice versa, how the model helps to better understand the ecological behaviour of the biological system.

## The Tilman model

The general form given by Tilman (1982), using his notation, for a system with an arbitrary number of consumer population densities $N_{i} \left( t \right)$ and resource abundances $R_{j} \left( t \right),$ the state variables of the system, is

1 $$\left\{ \begin{array}{ll} \frac{{dN_{i} (t)}}{dt} = f_{i} (R_{1} ,R_{2} , \ldots )N_{i} (t) - m_{i} N_{i} (t)\\ \frac{{dR_{j} (t)}}{dt} = g_{j} (R_{j} ) - \sum\limits_{i} {q_{ji} (R_{1} ,R_{2} , \ldots )f_{i} (R_{1} ,R_{2} , \ldots )N_{i} (t)}\\ \end{array} \right.$$

Here *m*_*i*_ is the inherent net mortality rate of the consumer *i*, the average probability per time unit that a single consumer of this species is removed from the population. The ensuing net expectation for the decline rate of the population density is the product of the mortality rate and the density itself. The mortality rate is a constant parameter, not depending on any other model system variable or parameter. Similarly the function *f*_*i*_ is the relative growth rate of consumer *i*. This rate only depends on the resource densities. In our general analysis we will make just a few assumptions for this growth function, but leave it further unspecified. In “Numerical model calculations” we use the common Holling type II response function. Symbols and their physical dimension are summarized in “List of symbols”.

The dynamical equation for the resources in (1), like that for the consumers, contains a growth and a decline term. The growth rate *g*_*j*_ of any given resource depends only on the resource density itself. Within this paper we will describe the net inflow of nutrients with the familiar chemostat model $g_{j} \left( {R_{j} } \right) = a_{j} \left( {s_{j} - R_{j} } \right)$. In the absence of consumers it describes restricted exponential growth of the resource density towards *s*_*j*_, the stable resource density, at a rate *a*_*j*_. The *a*_*j*_ and *s*_*j*_ are constants. In fact *a* is the dilution rate of the chemostat, and 1/*a* is the average residence time of the nutrient inside the system, defining a time scale of the process. A chemostat model may be appropriate in an experimental setup, and applies for instance to a lake with abiotic nutrients delivered and removed through inflowing and outflowing streams. Some aspects of the model, however, may be somewhat counter-intuitive. We will mention these later briefly, and discuss them more extensively in a separate paper.

Consumers use the resources for growth. This leads to the decline term in the equation for the resources. The total rate at which a given resource is consumed is the sum of the consumption rates of all consumers using that particular resource. The consumption rate of a resource by a consumer is proportional to the growth rate of that consumer, with a proportionality factor accounting for the conversion from resource to consumer. In the original model as proposed by Tilman the conversion factor *q*_*ji*_ can be a function of all resource densities, but not of the consumer densities. We will use constant positive conversion factors *q*_*ji*_ for resource *R*_*j*_ used by consumer *N*_*i*_. This implies that in order to produce a single consumer unit, a fixed amount of each resource is removed.

Further attributes of the ecological system can enter the model through the growth functions $f_{i} \left( {R_{ 1} , R_{ 2} , \ldots } \right)$. In our general stability analysis we assume the growth rate of a consumer to increase with increasing resource density. A larger availability of a resource will make life easier for the consumer, and a smaller investment in finding the resource is expressed in an increased number of siblings or a decreased probability of starvation. For the model the net effect is the same. Secondly we assume that there can be no growth in the absence of resources; a population cannot grow without a resource being present, but instead will die from want. Within the chemostat model for nutrient supply one must be cautious though, because a low nutrient concentration is associated with a high net nutrient inflow. If consumption is very effective, a low abundance can be combined with a large flow. In reality often consumers need to get hold of the resource in order to benefit from it. If that includes foraging for the resource, it needs to be present in some finite abundance, otherwise the investment in foraging behaviour does not exceed the gain in acquiring the resource. The holy grail is to find the perfect balance between simplicity and applicability of the model (Hilborn and Mangel 1997). We will take the growth rate of the consumers to be zero at zero resource abundance.

For the terminology and a short overview of the mathematical analysis method the reader is referred to “Appendix A: Dynamical systems”. In a stability analysis we first find the stationary or equilibrium states of the system of equations by solving the dynamical equations (1) for the case of zero net growth; all derivatives are identically zero. Note that we use the terms equilibrium and stationary state interchangeably. Instead of state we will also use the term point, as explained in “Appendix A”. Exactly at the equilibrium state all derivatives are zero, nothing changes and the systems is fully stationary. The question is what happens if the system is slightly perturbed, does it move back to its equilibrium, or do the perturbations grow, rendering the stationary state unstable? Linearization of the non-linear system of equations about the stationary state next yields the Jacobi matrix, the eigenvalues and eigenvectors of which matrix exactly tell us how the model system reacts to small perturbations near the stationary state. We present these calculations in much detail in “Appendix B: Mathematical details of the stability analysis”, so the reader may check the steps along the way, and reproduce those steps in case of a slightly modified model system. “Stability analysis of the Tilman model” will focus on the results of these calculations. Next to the stable equilibria or stationary states, we also investigate the unstable stationary states of the system, as these will turn out to play an important role in the transient dynamics of the system, how it develops towards the stable state. Finally we investigate this full dynamics of the examples treated in the next section for a specific class of growth functions. As the differential equations are non-linear, we use an approximating numerical solution procedure in “Numerical model calculations”.

## Stability analysis of the Tilman model

### A single species consuming a single resource

The simplest case of the Tilman model we investigate is that of a single consumer and a single resource. We adopt a simplified notation; instead of *N*_1_ we use *B* (a second consumer *A* is introduced later), instead of *R*_1_ we write *R* (and a second resource is named *P*). The model equations for a single species *B* depending on a single resource *R* are

2a $$\frac{dB(t)}{dt} = f_{B} (R(t))B(t) - m_{B} B(t) ,$$

2b $$\frac{dR(t)}{dt} = a_{R} (s_{R} - R(t)) - q_{RB} f_{B} (R(t))B(t).$$

Here *f*_*B*_(*R*(*t*)) is the resource dependent relative growth rate of *B*, and *m*_*B*_ is its mortality. We perform the stability analysis with a general function *f*, because we want to show that for the chosen characteristics of this function the stability of the steady state is the same, irrespective of the exact form of *f*. In the absence of *B* the resource *R* is depleted at a rate *a*_*R*_, while it is replenished to a stable level *s*_*R*_. When *B* is present, the resource is additionally depleted at the same rate at which *B* grows, times a conversion factor *q*_*RB*_. For this simple system we will do the mathematical derivation in full here, derivations for all systems we consider are given in “Appendix B”. We first determine the stationary states (or points) of the system and their stability properties.

From Eq. (2a) it follows directly that if there are no *B*’s to begin with, there will never be any. Moreover, in the absence of *B*, the resource will asymptotically reach a level *s*_*R*_, regardless of the initial level *R*(0). This implies

3 $$B_{0} = 0,\;\;R_{0} = s_{R} ,$$

is one of the stationary points of the system of equations (2a, 2b). We will call this the trivial equilibrium, because a key element of the model, i.e. the consumer, is absent. The parameter *s*_*R*_ we call the stable resource level.

To investigate the stability of the trivial equilibrium in the full non-linear system, we determine the eigenvalues of the Jacobi matrix at the equilibrium point. An explanation of the derivation is given in “Appendix A”. The Jacobi matrix for system (2a, 2b) is given by

4 $$J(B,R) = \left( {\begin{array}{*{20}c} {f_{B} (R) - m_{B} } & {B\frac{{df_{B} (R)}}{dR}} \\ { - q_{RB} f_{B} (R)} & { - a_{R} - q_{RB} B\frac{{df_{B} (R)}}{dR}} \\ \end{array} } \right).$$

For the trivial equilibrium (3) we find

5 $$J(0,s_{R} ) = \left( {\begin{array}{*{20}c} {f_{B} (s_{R} ) - m_{B} } & 0 \\ { - q_{RB} f_{B} (s_{R} )} & { - a_{R} } \\ \end{array} } \right) ,$$

with eigenvalues $\lambda_{ 1} = f_{B} \left( {s_{R} } \right) - m_{B} \;{\text{and}}\;\lambda_{ 2} = - a_{R}$. Note that the eigenvalues have the dimension of a rate, a negative value gives the rate at which the system moves towards the stationary state, a positive one the rate at which it moves away. For any biologically relevant situation the parameter *a*_*R*_ is positive, so one of the eigenvalues is always negative. If the mortality is larger than *f*_*B*_(*s*_*R*_), the growth function at stable resource density, also the second eigenvalue is negative, and the stationary point (0, *s*_*R*_) is a stable node. If the mortality is smaller than *f*_*B*_(*s*_*R*_), the first eigenvalue is positive, so the stationary point is a saddle point, which is unstable (also see “Appendix A” for a characterisation of stationary states). If there is enough of the nutrient for the consumer to compensate its net mortality, any existing small consumer density will increase, otherwise the consumer disappears.

A second stationary point is found by solving (2a) for *dB*/*dt* = 0. Apart from *B* = 0, that upon substitution in (2b) gives the trivial stationary point, there is also a possible solution

6 $$f_{B} \left( R \right) = m_{B} .$$

In this case the resource concentration is such that growth exactly compensates for the loss term of the consumer, the net growth is zero. Whether there is such a solution depends on the details of *f*_*B*_(*R*). The growth function we have chosen is maximal for infinite resource density. If this maximum growth rate lies below the mortality rate of the consumer, then *f*_*B*_(*R*) < *m*_*B*_ for all values of *R*, and there is no solution of (6). In that case the trivial equilibrium (0, *s*_*R*_) is a stable node. In fact for any positive value of the resource and consumer density the system will develop towards the trivial equilibrium.

If the maximum of the growth function exceeds the mortality, there is exactly one solution $f_{B} \left( {R {\kern 1pt} ^{*} } \right) = m_{B}$ of Eq. (6), because the growth function is monotonously increasing with increasing resource density. The corresponding equilibrium density $B{\kern 1pt} ^*$ then is found by setting *dR*/*dt* = 0 in (2b). This leads to

7 $$B {\kern 1pt} ^* = \frac{{a_{R} (s_{R} - R{\kern 1pt} ^*)}}{{q_{RB} f_{B} (R{\kern 1pt} ^*)}} = \frac{{a_{R} (s_{R} - R^*)}}{{q_{RB} m_{B} }} .$$

This implies there is a second stationary point of the full system

8 $$B{\kern 1pt}^* = \frac{{a_{R} (s_{R} - R{\kern 1pt}^*)}}{{q_{RB} m_{B} }},\;\;R = R{\kern 1pt} ^* ,$$

describing coexistence of the consumer and the resource. We call this the coexistence point. Now there are two possibilities: if $R{\kern 1pt} ^* > s_{R}$ the equilibrium density for the consumer in the coexistence point is negative, which is biologically impossible. In this case the trivial stationary point is a stable node; the consumer will always die out, and the resource will reach its stable level. The second possibility is that $R{\kern 1pt} ^* < s_{R}$, in which case the coexistence point is biologically relevant, and the trivial equilibrium is a saddle point.

The Jacobi matrix for the coexistence point (8) is

9 $$J(B{\kern 1pt} ^*,R{\kern 1pt} ^*) = \left( {\begin{array}{*{20}c} 0 & {B{\kern 1pt} ^*\left. {\frac{{df_{B} (R)}}{dR}} \right|_{R = R{\kern 1pt} ^*} } \\ { - q_{RB} m_{B} } & { - a_{R} - q_{RB} B{\kern 1pt} ^*\left. {\frac{{df_{B} (R)}}{dR}} \right|_{R = R{\kern 1pt} ^*} } \\ \end{array} } \right).$$

The eigenvalues are

10 $$\lambda_{ \pm } = - \tfrac{1}{2}a_{R} - \tfrac{1}{2}q_{RB} B{\kern 1pt} ^*{\kern 1pt} f^{\prime}_{B} (R{\kern 1pt} ^*) \pm \tfrac{1}{2}\sqrt {(a_{R} + q_{RB} B{\kern 1pt} ^*{\kern 1pt} f^{\prime}_{B} (R*))^{2} - 4m_{B} q_{RB} B{\kern 1pt} ^*{\kern 1pt} f^{\prime}_{B} (R*)} .$$

If the coexistence point is biologically relevant, both eigenvalues have a negative real part, and the equilibrium is a stable node or a stable vortex. Only if the stable resource level is more than sufficient for the consumer to overcome its inherent mortality, there is a stable finite size population. In “One consumer and one resource” we investigate the full dynamics for a Holling type II growth function. Further discussion of the ecological implications of these results is deferred to the “Conclusions and discussion”.

### Two species competing for a single resource

When two different species *A* and *B* are competing for the same resource *R*, the set of equations is extended with an equation for *A* analogous to that for *B*, while the consumption of the resource by both species now is included in the equation for *R*. We have

11a $$\frac{dA(t)}{dt} = f_{A} (R)A(t) - m_{A} A(t) ,$$

11b $$\frac{dB(t)}{dt} = f_{B} (R)B(t) - m_{B} B(t) ,$$

11c $$\frac{dR(t)}{dt} = a_{R} (s_{R} - R(t)) - q_{RA} f_{A} (R(t))A(t) - q_{RB} f_{B} (R(t))B(t).$$

Again the resource reaches a stable level *s*_*R*_ in the absence of *A* and *B*, establishing the trivial equilibrium

12 $$A = 0,\;\;B = 0,\;\;R = s_{R} .$$

A second stationary point is found by solving (11a) for zero growth, to find

13 $$f_{A} (R) = m_{A} ,$$

while (11b) is satisfied because *B* = 0; one consumer is absent. The analysis is fully in line with that for a single species. Again the details of the growth function determine whether a solution to (13) exists. There is at most one solution $R_{A}^{*}$. The associated equilibrium density A*  is derived from (11c) by setting *dR*/*dt* = 0. The second equilibrium, the *A*-point, is

14 $$A{\kern 1pt} ^* = \frac{{a_{R} (s_{R} - R_{A}^{*} )}}{{q_{RA} m_{A} }},\;\;B = 0,\;\;R = R_{A}^{*} .$$

A similar third stationary point is found when

15 $$f_{B} (R) = m_{B} .$$

and *A* = 0. The third equilibrium, the *B*-point, is

16 $$A = 0,\;\;B{\kern 1pt} ^* = \frac{{a_{R} (s_{R} - R_{B}^{*} )}}{{q_{RB} m_{B} }},\;\;R = R_{B}^{*} .$$

Stationary points (14) and (16) are only biologically relevant if $s_{R} > R_{A}^{*}$ or $s_{R} > R_{B}^{*}$ respectively, that is, when the stable resource level exceeds the required level for net population growth of the consumer species.

The next question is about the stability of the three equilibria as a function of the stable resource level *s*_*R*_. We assume that $R_{A}^{*} < R_{B}^{*}$. After all, the names are just conventional. If $s_{R} > R_{A}^{*}$ (and hence also $s_{R} > R_{B}^{*}$), there is not enough resource to sustain any consumer. Both (14) and (16) then are unphysical, if they exist at all, because a density cannot be negative. The only real equilibrium is the trivial one, which is a stable node (see “Appendix B: Two species competing for a single resource”). The next case is when $R_{A}^{*} < s_{R} < R_{B}^{*}$, so there is enough resource for *A* to grow to its stable level A*, which now is a positive number. Equilibrium (16) still is unphysical. The trivial equilibrium (12) is a saddle point, and equilibrium (14) is a stable node or a stable vortex. Finally we can have $R_{A}^{*} < R_{B}^{*} < s_{R}$, in which case all three equilibria are biologically relevant. The trivial equilibrium then is a saddle. Equilibrium (14) still is a stable node or a stable vortex, and (16) is a saddle point.

There seem to be only two possibilities. If the steady nutrient supply is insufficient to sustain either consumers, both species become extinct and the resource reaches its stable level; the system goes to the trivial equilibrium. The alternative is that the species with the lower food requirement reaches a stable level, the other becomes extinct and one of the border equilibria is reached. Close to the equilibrium point this is true, but we cannot draw global conclusions from this analysis. We do not have any information about the actual dynamics of the system away from the stationary points. Numerical investigation for specific parameter values and growth functions, as we will do in “Numerical model calculations: two consumers and one resource”, will show this global behaviour in detail, and will show that in fact unstable points can be extremely relevant for what happens in real systems. The conclusion for now is that there can be coexistence between one consumer and one resource, but a single resource in the long run is insufficient, within the model we study, to sustain two different consumers in stable coexistence.

### A single species consuming two resources

For a single species depending on two different resources, the dynamical equations become

17a $$\frac{dB(t)}{dt} = f_{B} (P(t),R(t))B(t) - m_{B} B(t) ,$$

17b $$\frac{dP(t)}{dt} = a_{P} (s_{P} - P(t)) - q_{PB} f_{B} (P(t),R(t))B(t) ,$$

17c $$\frac{dR(t)}{dt} = a_{R} (s_{R} - R(t)) - q_{RB} f_{B} (P(t),R(t))B(t) .$$

Note that the conversion factors *q*_*PB*_ and *q*_*RB*_ can be different for the two resources. Also the rates *a*_*P*_ and *a*_*R*_ can be different for the two resources, in case of a chemostat with a single supply reservoir the dilution rates usually will be the same. The growth rate of the species depends on both abundances, allowing for a trade-off, at least that seems the case. If both resources are needed, $f_{B} \left( {P, R} \right)$ will be zero in the absence of either resource.

The coupling between the two resources is strictly through the consumption by *B*. Both have a stable replenishing level, different for different resources, and independent of the other resource. In the absence of *B*, there will never be any, and both resources are replenished independently to their stable levels, regardless of their initial abundance, so there is a stationary point

18 $$B = 0,\;\;P = s_{P} ,\;\;R = s_{R} .$$

As before, we will call this the trivial equilibrium. The point with both resources at their stable levels is called the supply point.

Zero net growth for *B* is also the case if the growth rate matches its mortality rate

19 $$f_{B} (P,R) = m_{B} .$$

Whether (19) has solutions depends on the growth function. We must be a bit more specific now we have two resources. We expect a higher abundance of either resource to give a higher growth potential. Hence we assume a monotonically non-decreasing function $f_{B} \left( {P, R} \right)$, so $\partial f/\partial P \ge 0$ and $\partial f/\partial R \ge 0$ regardless of the abundances of the resources. Those assumptions still leave open all kinds of interactions, like substitutability or synergy between the resources in the consumption pattern, but rules out inhibition, where a high abundance of one or both resources reduces the growth.

The solution of (19) is a contour line of $f_{B} \left( {P, R} \right)$ in the *PR*-plane at the value *m*_*B*_, called the zero growth isocline of *B*. If $f_{B} \left( {P, R} \right) < m_{B}$ for all abundances, there is no solution. Otherwise, in general, the zero growth isocline gives infinitely many combinations of resource abundances for which (17a) yields a stationary *B* population size. In the case there are no solutions to (19), the trivial equilibrium is the only stationary point. If there are infinitely many solutions to (19) a second criterion comes from the fact that the same stable *B*-density should satisfy both (17b) and (17c).

Suppose (*P*, *R*) is a solution of (19), any point along the contour line. In order for this specific combination of resource densities to establish a stationary point, both (17b) and (17c) need to give zero growth. That leads to the set of linear equations

20 $$\left\{ \begin{aligned} a_{P} (s_{P} - P) - q_{PB} m_{B} B = 0 \hfill \\ a_{R} (s_{R} - R) - q_{RB} m_{B} B = 0 \hfill \\ \end{aligned} \right..$$

Since the same population density *B* occurs in both equations, it can be eliminated to give

21 $$\frac{{a_{P} (s_{P} - P)}}{{q_{PB} m_{B} }} = \frac{{a_{R} (s_{R} - R)}}{{q_{RB} m_{B} }}\;\; \Rightarrow \;\;\frac{{s_{P} - P}}{{s_{R} - R}} = \frac{{a_{R} q_{PB} }}{{a_{P} q_{RB} }} .$$

This identifies a line in the *PR*-plane through *P* = *s*_*P*_, *R* = *s*_*R*_, the supply point, with the slope given by the *q*’s and the *a*’s. The stationary point is the intersection (P*, R*) of this line with the *m*_*B*_-contour of *f*_*B*_(*P*, *R*). If both P* and R* are above the corresponding stable level for the given resource, the population size B* is unphysical, so only for both values below the stable level do we find a biologically relevant second stationary point.

22 $$B{\kern 1pt} ^* = \frac{{a_{R} (s_{R} - R{\kern 1pt} ^*)}}{{q_{RB} m_{B} }} = \frac{{a_{P} (s_{P} - P{\kern 1pt} *)}}{{q_{PB} m_{B} }},\;P = P{\kern 1pt} ^*,\;R = R{\kern 1pt} ^*.$$

As before, we will call this the coexistence point.

In “Appendix B: A single species consuming two different resources” we show that if there is a biologically relevant coexistence point the trivial equilibrium is a saddle point, otherwise it is a stable node. If the amount of resources made available is insufficient to compensate for the mortality, the species will become extinct. It looks like the introduction of a second resource does not add to the complexity of the biological system, it only complicates the mathematics. Having a second resource available does not provide the consumer with an option to exchange between the two, its behaviour is fixed by how the growth function depends on the two resource densities and the fixed values of the other parameters.

### Two species competing for two resources

So far we have seen that within the model there can be sustainable coexistence between the food and the consumer and between two foods and one consumer, but not between two consumers and one food. Can we have coexistence between two consumers and if so, under what circumstances? We look at a system with two consumers and two resources

23a $$\frac{dA(t)}{dt} = f_{A} (P(t),R(t))A(t) - m_{A} A(t),$$

23b $$\frac{dB(t)}{dt} = f_{B} (P(t),R(t))B(t) - m_{B} B(t),$$

23c $$\frac{dP(t)}{dt} = a_{P} (s_{P} - P(t)) - q_{PA} f_{A} (P(t),R(t))A(t) - q_{PB} f_{B} (P(t),R(t))B(t),$$

23d $$\frac{dR(t)}{dt} = a_{R} (s_{R} - R(t)) - q_{RA} f_{A} (P(t),R(t))A(t) - q_{RB} f_{B} (P(t),R(t))B(t).$$

There are four stationary points. First we have the trivial equilibrium, where both *A* and *B* are absent

24 $$A = 0,\;\;B = 0,\;\;P = s_{P} ,\;\;R = s_{R} .$$

Next we have the situation where *B* is absent and *A* is stable because

25 $$f_{A} (P_{A}^{*} ,R_{A}^{*} ) = m_{A} ,$$

that is the growth matches the mortality, a point on the *m*_*A*_-contour line or null isocline for *A*. As for a single species with two foods, we have similar to Eq. (18)

26 $$\frac{{s_{P} - P_{A}^{*} }}{{s_{R} - R_{A}^{*} }} = \frac{{a_{R} q_{PA} }}{{a_{P} q_{RA} }}.$$

The stationary point is

27 $$A^{\prime} = \frac{{a_{R} (s_{R} - R_{A}^{*} )}}{{q_{RA} m_{A} }} = \frac{{a_{P} (s_{P} - P_{A}^{*} )}}{{q_{PA} m_{A} }},\;\;B = 0,\;\;P = P_{A}^{*} ,\;\;R = R_{A}^{*} .$$

Whether this stationary point is biologically relevant depends on the growth function and the stable resource levels. We will call this the *A*-point. Reversely we may have that *A* is absent while for *B*

28 $$f_{B} (P_{B}^{*} ,R_{B}^{*} ) = m_{B} ,$$

with

29 $$\frac{{s_{P} - P_{B}^{*} }}{{s_{R} - R_{B}^{*} }} = \frac{{a_{R} q_{PB} }}{{a_{P} q_{RB} }},$$

to give the stationary *B*-point

30 $$A = 0,\;\;B^{\prime} = \frac{{a_{R} (s_{R} - R_{B}^{*} )}}{{q_{RB} m_{B} }} = \frac{{a_{P} (s_{P} - P_{B}^{*} )}}{{q_{PB} m_{B} }},\;\;P = P_{B}^{*} ,\;\;R = R_{B}^{*} .$$

Again the specifics of the growth function and the resources determine the biological relevance. A fourth stationary point indeed sees the coexistence of all four state variables. When the null isoclines (25) and (28) intersect, there is a combination of resource abundances

31 $$\left\{ \begin{array}{l} f_{A} (P{\kern 1pt} ^*,R{\kern 1pt} ^*) = m_{A}\\ f_{B} (P{\kern 1pt} ^*,R{\kern 1pt} ^*) = m_{B} \\ \end{array} \right..$$

Because also (23c) and (23d) need to show zero change we have

32 $$\left\{ \begin{array}{l} a_{P} (s_{P} - P{\kern 1pt} ^*) - q_{PA} m_{A} A - q_{PB} m_{B} B = 0 \\ a_{R} (s_{R} - R{\kern 1pt} ^*) - q_{RA} m_{A} A - q_{RB} m_{B} B = 0\\ \end{array} \right..$$

This linear system can be written in matrix–vector form as:

33 $$Q\;\left( {\begin{array}{*{20}c} {m_{A} A} \\ {m_{B} B} \\ \end{array} } \right) = \left( {\begin{array}{*{20}c} {a_{P} (s_{P} - P{\kern 1pt} ^*)} \\ {a_{R} (s_{R} - R{\kern 1pt} ^*)} \\ \end{array} } \right)\;,\;\;\;\;{\text{with}}\;\;\;\;Q = \left( {\begin{array}{*{20}c} {q_{PA} } & {q_{PB} } \\ {q_{RA} } & {q_{RB} } \\ \end{array} } \right).$$

If this matrix *Q* has an inverse, this system can be solved for the population sizes to give

34 $$A ^* = \frac{{q_{RB} a_{P} (s_{P} - P{\kern 1pt} ^*) - q_{PB} a_{R} (s_{R} - R{\kern 1pt} ^*)}}{{(q_{PA} q_{RB} - q_{RA} q_{PB} )\;m_{A} }},\;\;B{\kern 1pt} ^* = \frac{{q_{PA} a_{R} (s_{R} - R{\kern 1pt} ^*) - q_{RA} a_{P} (s_{P} - P{\kern 1pt} ^*)}}{{(q_{PA} q_{RB} - q_{RA} q_{PB} )\;m_{B} }}$$

In order for the equilibrium to be biologically relevant, both population sizes must be positive. It is not enough for the stable resource levels to exceed the stationary levels here, depending on the determinant of the *Q*-matrix the parameters must satisfy

35 $$\frac{{q_{PB} }}{{q_{RB} }} < \frac{{a_{P} (s_{P} - P{\kern 1pt} ^*)}}{{a_{R} (s_{R} - R{\kern 1pt} ^*)}} < \frac{{q_{PA} }}{{q_{RA} }},$$

for det (*Q*) > 0, or

36 $$\frac{{q_{PB} }}{{q_{RB} }} > \frac{{a_{P} (s_{P} - P{\kern 1pt} ^*)}}{{a_{R} (s_{R} - R{\kern 1pt} ^*)}} > \frac{{q_{PA} }}{{q_{RA} }},$$

for det (*Q*) < 0. If either is the case, Eqs. (31) and (34) establish a fourth equilibrium, with coexistence of all consumers and resources

37 $$A = A ^*,\;\;B = B{\kern 1pt} ^*,\;\;P = P{\kern 1pt} ^*,\;\;R = R{\kern 1pt} ^* .$$

If there are more intersection points satisfying (31) there can be additional equilibria of the same type. The inequalities (35) and (36) are related to the usual graphical analysis of the Tilman model in the *PR*-plane (Tilman 1980; also see Ballyk and Wolkowicz 2011, for a detailed description of a slightly different approach). For the case that the *a*’s are the same, a common choice for a chemostat, both inequalities state that the supply point lies in the wedge between the lines through (P*, R*), with slopes given by the ratios of the conversion factors. The direction vectors of these lines are called the consumption vectors.

Stability analysis (see “Appendix B: Two species and two resources”) shows that if the stable level of the resources is sufficient to overcome the mortality of at least one of the consumers, the trivial equilibrium is a saddle point, otherwise it is a stable node. The *A*-point and *B*-point behave as for a single consumer with two resources. The stability of the coexistence point is related to

38 $$\det \left( {\begin{array}{*{20}c} {q_{PA} } & {q_{PB} } \\ {q_{RA} } & {q_{RB} } \\ \end{array} } \right)\det \left( {\begin{array}{*{20}c} {\frac{{\partial f_{A} }}{\partial P}} & {\frac{{\partial f_{A} }}{\partial R}} \\ {\frac{{\partial f_{B} }}{\partial P}} & {\frac{{\partial f_{B} }}{\partial R}} \\ \end{array} } \right).$$

The *Q*-matrix is already familiar. It plays a role in determining the biological relevance of the coexistence point. The derivatives in the second matrix are evaluated at the intersection point of the null isoclines. The columns are the gradients of the growth functions, which vectors are perpendicular to the contour lines. The determinant indicates how these contour lines cross. If the determinants have opposite sign, we have a saddle point, otherwise it is a stable node or stable vortex.

**Fig. 1:**
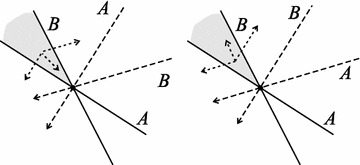
Stable or unstable coexistence of two species *A* and *B* in the *PR*-plane. The *drawn lines* are the zero isoclines. At the intersection point both consumer densities are stationary. The *dashed arrows* are consumption vectors, indicating in what proportion the two resource densities decrease when a single consumer density increases. The *dotted arrows* in the shaded area give the combined resource density change; a motion towards the intersection, and hence a stable equilibrium point (*left*), or away from it, and hence a saddle point (*right*)

In Fig. 1 this is elucidated in the *PR*-plane. Note that the full dynamics, as given in the analysis above, is in a four dimensional phase space, but because of the structure of the equations and with some additional notions one may investigate the behaviour in the projection plane only. The drawn lines marked *A* and *B* are the zero isoclines for each consumer, the dashed arrows have a slope corresponding to the uptake ratio of the resources of the consumer as indicated. These consumption vectors indicate how the resource concentrations change if the consumer density changes. The determinant of *Q* tells us how the uptake vectors cross, whether you rotate clockwise moving from *A* to *B* or anticlockwise. In the left plot the uptake vectors cross in the same way as the zero isoclines, both clockwise, the determinants of the two matrices have the same sign, and we have a stable equilibrium. In the right plot the crossing is reversed, the uptake vectors cross anticlockwise, and we have a saddle point. This can further be understood by looking at a point in the shaded wedge between the two isoclines, close to the stationary coexistence point. In both cases it lies to the left of the *B*-isocline, which means there are not enough nutrients to compensate for its mortality, so *B* will decrease. Consequently, according to the dynamical equations, *P* and *R* will increase in proportions given by the *B*’s conversion factors, opposite to the uptake vector for *B*. The shaded area is to the right of the *A*-isocline, so *A* will increase and the resources will decrease in the same direction as the uptake vector for *A*. The dotted arrows indicate both changes. In the left case the combined arrow, the sum of the two effects, points towards the intersection point, in the right case it points away from it. One may repeat the procedure in the other wedges to ascertain that indeed the left case is a stable equilibrium and the right case a saddle, as indicated by the mathematical analysis. A more popular statement is that for stable coexistence of the consumers not only the supply of the resource must suffice, but also each consumer should consume mostly that resource that in the intersection point is mostly limiting its growth.

## Numerical model calculations

For a specific model we can readily calculate the full solution of the dynamic equations numerically. A standard forward Euler approximation (Press et al. 1986) with a sufficiently small time integration step will usually perform well. We have used a rather straightforward implementation of the model in Excel. We again consider the four different situations, single consumer with single resource, two consumers and a single resource, single consumer and two resources and two consumers with two resources. For each of the systems we calculate the stationary points and their stability properties as a function of the stable level of the resource(s). Next we investigate the global behaviour by following the trajectory of the system in an appropriate part of phase space by numerically integrating the full non-linear system of differential equations for an appropriately chosen initial situation.

### One consumer and one resource

For the growth function of the consumer we now take the Holling type II functional response

39 $$f_{B} (R) = \frac{{f_{mB} R}}{{R + k_{RB} }},$$

The solution of (6) for this simple case can be calculated analytically

40 $$f_{B} (R{\kern 1pt} ^*) = \frac{{f_{mB} R}}{{R + k_{RB} }} = m_{B} \;\; \Rightarrow \;\;R{\kern 1pt} ^* = \frac{{k_{RB} m_{B} }}{{f_{mB} - m_{B} }} .$$

The solution only exists if the maximum *f*_*mB*_ of the growth function exceeds the mortality rate *m*_*B*_. Moreover for the coexistence point (10) to form a stable attractor in the positive quadrant, $R{\kern 1pt} ^* < s_{R}$, so

41 $$\frac{{k_{RB} m_{B} }}{{f_{mB} - m_{B} }} < s_{R} \;\; \Rightarrow \;\;f_{mB} > m_{B} \;\left( {1 + \frac{{k_{RB} }}{{s_{R} }}} \right) .$$

The half saturation constant *k*_*RB*_ mainly sets the scale for *s*_*R*_, and hence the resource density *R*. Similarly *q*_*RB*_ sets the relative scale of the consumer density *B* in Eq. (2b). The mortality rate *m*_*B*_ sets a time scale for the consumer dynamics, while *a*_*R*_ does the same for the resource. We take *k*_*RB*_ = 1. If the coexistence point is biologically relevant, the trivial equilibrium at zero consumer density is a saddle point, otherwise it is a stable node.

**Fig. 2:**
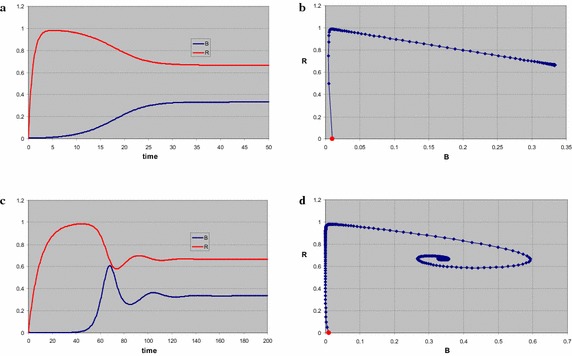
Numerical solution of a system of a single consumer *B* and a single resource *R* (parameter values, specified in the text, are such that there is stable coexistence of consumer and resource). Initially the resource is absent and the consumer density is small. The time plot **a** shows that the resource rapidly grows to its stable level, while the consumer density remains small. When the latter increases, the resource density drops, and both densities relax to the coexistence equilibrium level. The phase plot **b** shows the trajectory as produced by the consecutive states of the numerical iteration procedure with fixed time step, the marker points. The system moves rapidly from the initial state, indicated by the *red dot*, to the trivial equilibrium, a saddle point, and next moves directly to the stable coexistence point. Next the parameters are modified to create a substantial difference in time scales between the growth rates of the resource and the consumer. The time plot **c** shows that in the final relaxation both densities show oscillating behaviour. Again the phase plot **d** shows that the system first moves to the unstable trivial equilibrium, but now spirals into the stable coexistence point

Figure 2a, b shows the results for the case *m*_*B*_ = 1, *a*_*R*_ = 1, *s*_*R*_ = 1, *q*_*RB*_ = 1. According to (41) the maximum of the growth function must be *f*_*mB*_ > 2 to have a stable coexistence point. We take *f*_*mB*_ = 2.5. The starting point of the numerical solution of the system of equations we take at *R*(0) = 0, *B*(0) = 0.01, no resource and just a small consumer density. The time plot (Fig. 2a) shows that the resource density quickly grows to its stable level, while the consumer density remains small. Once the consumer density starts increasing, the resource density drops until stable coexistence of the consumer and its resource is reached at R* = 0.67, B* = 0.33. In the phase plot (Fig. 2b) the consecutive time steps are marked, showing that the consumer density initially drops rapidly, as can be expected in the absence of food, until the dynamics slows down near the trivial equilibrium at *R* = 1, *B* = 0. Since this is a saddle point, with eigenvalues *λ*_1_ = 0.250 and *λ*_2_ = −1, the trajectory eventually accelerates along the unstable direction, straight to the stable coexistence point, a stable node with eigenvalues *λ*_1_ = −0.300 and *λ*_2_ = −1. The eigenvalues at the saddle point are *λ*_1_ = *f*_*B*_(*s*_*R*_) − *m*_*B*_ and *λ*_2_ = −*a*_*R*_. The first one is always positive if the nutrient supply is sufficient, but the rate at which the system moves away from the saddle point can be very small. If we take 2 < *f*_*mB*_ < 2.5; smaller, but large enough to feed *B*, the system initially moves very rapidly towards the saddle point, because of the second eigenvalue *λ*_2_ = −1, but moves away at an arbitrarily small rate.

If the timescale for the resource replenishment is chosen substantially larger than that of the consumer mortality, i.e. slow replenishment, the coexistence point is a stable vortex. For *m*_*B*_ = 1, *a*_*R*_ = 0.1, *s*_*R*_ = 1, and *q*_*RB*_ = 0.1, the two stationary points have exactly the same density values as above, but the dynamics is different. The initial values are also the same: *R*(0) = 0, *B*(0) = 0.01. Again in the phase plot (Fig. 2d) the system moves towards the trivial equilibrium, lingers there until it speeds up in the unstable direction, but then spirals into the coexistence point. Note the difference in time scale with the previous situation in the time series (Fig. 2c). Once the consumer density starts growing, the resource density drops, but both overshoot their stable value. The relaxation towards the coexistence point shows oscillatory behaviour, and is also slower than in the previous case, but the reduction factor is not as high as that for the *a*_*R*_. The eigenvalues form a complex pair with negative real part Re(*λ*) = −0.065. The difference with the *λ*_1_ of the previous case is less than a factor of 5.

### Two consumers and one resource

With two consumers, there are two (different) growth functions

42 $$f_{A} (R) = \frac{{f_{mA} R}}{{R + k_{RA} }},\quad f_{B} (R) = \frac{{f_{mB} R}}{{R + k_{RB} }}.$$

This leads to the two critical resource levels

43 $$R_{A}^{*} = \frac{{k_{RA} m_{A} }}{{f_{mA} - m_{A} }},\quad R_{B}^{*} = \frac{{k_{RB} m_{B} }}{{f_{mB} - m_{B} }}.$$

The two cases of interest are *R*_A_^*^ < *s*_*R*_ < *R*_B_^*^, and *R*_A_^*^ < *R*_B_^*^ < *s*_*R*_. For the resource we take *a*_*R*_ = 1, and we look what the dynamics of the system is as a function of the stable level *s*_*R*_. The scale factors in both growth functions we take unity, the only difference between the consumers is in the maximal value of the growth function. For *A* we take *m*_*A*_ = 1, *q*_*RA*_ = 1,  $k_{RA} {\kern 1pt} = 1,$ and *f*_*mA*_ = 3, so *R*_A_^*^ = 0.5, while for *B* we take *m*_*B*_ = 1, *q*_*RB*_ = 1,  $k_{RB} {\kern 1pt} = 1$, and *f*_*mB*_ = 2, so *R*_B_^*^ = 1. *A* has the advantage, as will be confirmed shortly. For initial state we take *R*(0) = 0,  *A*(0) = 0.001,  and *B*(0) = 1, so we investigate whether indeed *A* takes over from *B*.

**Fig. 3:**
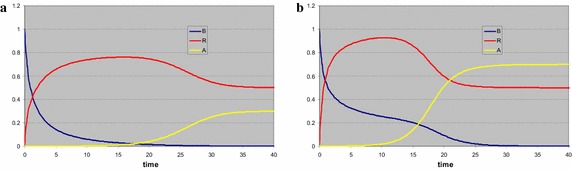
Two consumers and a single resource. Parameters, given in the text, are the same for both plots, the only difference is the supply level of the resource. Also the starting point is the same, no food, much of *B* and a little bit of *A*. In both cases *A* successfully takes over from *B*. If the stable resource level is below the critical level for maintenance of *B* (**a**), this consumer simply disappears, and at some later time *A* grows to its stationary level. If the supply of resource is sufficient to support *B* (**b**), there is an interval where a finite population *B* survives on the available resource. The decline of the species *B* is in fact brought about by its competitor *A* eating away the required food. Eventually a higher population of *A* is reached because of a higher supply

For *s*_*R*_ < 0.5 the only biologically relevant stationary point is the trivial equilibrium, which is a stable node. Any initial state will evolve towards it, like for a single consumer. Both *A* and *B* die out, and finally *R* grows to its stable level. For *s*_*R*_ = 0.8 (Fig. 3a), *B* dies out, be it rather slowly, *R* grows to its stable level, after which *A* picks up and grows to its stationary level, while the resource density drops. The trivial equilibrium is a saddle point, the coexistence of *A* and *R* is a stable node. For *s*_*R*_ = 1.2 (Fig. 3b), *R* grows to a value slightly below unity, while *B* decreases to about the stationary level *B* * = 0.2, but eventually *A* takes over. The coexistence of *B* and *R* is a saddle point, the coexistence of *A* and *R* is a stable node. The size of the stable population of *A* is higher for the higher stable resource level, while the stationary resource level in the latter two plots is exactly the same. The reason is that although in the stationary point the abundance of the resource is the same, because of the higher stable level, the production rate of the resource is higher. Hence a higher consumer population level can be maintained.

Again the devil is in the details. In any case consumer *A* eventually takes over from *B*, but it depends on the specific parameter values at which rate the system moves away from the trivial saddle or the unstable equilibrium for *B*. For instance if the advantage of *A* over *B* is substantially less than in the example in Fig. 3b above, for all practical purposes the saddle may appear to be stable, simply because the (numerical or real) experiment does not last long enough. Moreover, if the difference between the two consumers is relatively small, also the takeover away from the stationary points is very slow.

### One consumer and two resources

The growth function for a single species consuming two different resources is

44 $$f_{B} (P,R) = f_{mB} \hbox{min} \left( {\frac{P}{{P + k_{PB} }},\frac{R}{{R + k_{RB} }}} \right),$$

implying that for a given growth rate both resources need to have a certain minimal level; both *P* and *R* are essential resources. The zero growth isocline for *B* is given by two semi lines, parallel to the *P*-axis and *R*-axis, and starting in (*P*_*B*_, *R*_*B*_)

45 $$f_{B} (P,R) = m_{B} \;\; \Rightarrow \;\;\left( {P = P_{B} = \frac{{k_{PB} m_{B} }}{{f_{mB} - m_{B} }},R > R_{B} } \right),\;\;\left( {P > P_{B} ,R = R_{B} = \frac{{k_{RB} m_{B} }}{{f_{mB} - m_{B} }}} \right).$$

In order that the consumer population at equilibrium is positive, we must have *s*_*P*_ > *P*_*B*_ and *s*_*R*_ > *R*_*B*_. The intersection point is on one of the semi lines, hence either

46a $$P{\kern 1pt} ^* = \frac{{k_{PB} m_{B} }}{{f_{mB} - m_{B} }},\;\;R{\kern 1pt} ^* = s_{R} - \frac{{a_{P} q_{RB} }}{{a_{R} q_{PB} }}(s_{P} - P{\kern 1pt} ^*),\;\;$$

or

46b $$R{\kern 1pt} ^* = \frac{{k_{RB} m_{B} }}{{f_{mB} - m_{B} }},\;\;P{\kern 1pt} ^* = s_{P} - \frac{{a_{R} q_{PB} }}{{a_{P} q_{RB} }}(s_{R} - R{\kern 1pt} ^*),\;\;$$

depending on which semi line contains the intersection point. The stationary point is a stable node or stable vortex. We choose parameter values such that the intersection point is along the *P*_*B*_-semi line (*P* = *P*_*B*_, *R* > *R*_*B*_).

There are only two stationary points, the trivial one where the consumer is absent, and the coexistence point where there is a finite consumer population and both resources are present. If the stable level of either resource falls below the minimal required level, the system moves towards the trivial equilibrium, otherwise it moves towards the coexistence point. For the consumer we set $m_{B} {\kern 1pt} = 1$, $q_{PB} {\kern 1pt} = 1$, $q_{RB} {\kern 1pt} = 1$, and *f*_*mB*_ = 3, for the resources we set *a*_*P*_ = 1, *a*_*R*_ = 1, $k_{PB} {\kern 1pt} = 1$, $k_{PR} {\kern 1pt} = 1$, *s*_*R*_ = 1, and we investigate the behaviour of the system as a function of *s*_*P*_. The minimal required resource levels according to the above parameter values are *P*_0_ = 0.5 and *R*_0_ = 0.5, so the stable level for *R* is sufficient. We start at *P*(0) = 0,  *R*(0) = 0.

**Fig. 4:**
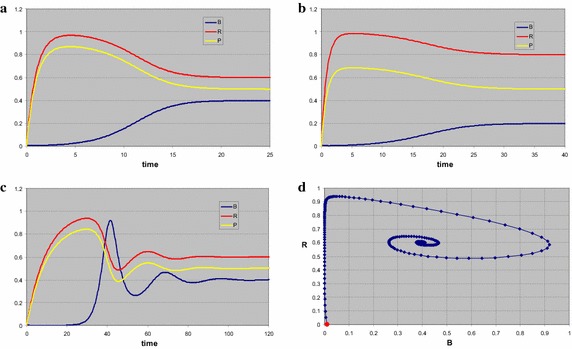
A single consumer and two essential resources. In all cases the system first develops towards the trivial equilibrium, which is unstable. Once the consumer density starts increasing, both resource densities are lowered. The difference between the simulations **a**, **b** is the stable resource level of *P*, which is the limiting resource. A lower supply level in **b** reduces the supply rate of this resource, and hence the stationary level of the consumer. The stationary level of *P* is exactly the same. Since less of *R* is consumed, but the supply remains the same, it reaches a higher stationary level. In **c**, **d** the coexistence point is exactly the same as in (**a**), but the consumer death rate and resource growth rates are quite different. Now the coexistence point is a stable vortex, with the trajectory spiralling towards it. The time plot shows oscillating behaviour

For *s*_*P*_ = 0.9, where we take as initial consumer density *B*(0) = 0.01, the system first moves towards the trivial equilibrium (Fig. 4a). Since this forms a saddle point here, once the consumer density starts building up, the resource abundances drop and we reach coexistence. For *s*_*P*_ = 0.7 the behaviour is very similar, be it that both equilibria have shifted (Fig. 4b). *P* reaches a lower maximum, in agreement with the lower stable level, but eventually drops to the same minimal level of 0.5. *R*, on the other hand, reaches the same maximum, but eventually reaches a higher abundance than in the previous case. The stable consumer density is lower. This is a rather complicated trade-off. Since the stable level for *P* is lowered, the production of that resource is lowered as well, so in order to have a stationary level, the consumer density is lowered. A lower number of consumers eats less *R*, so the stationary level of that resource increases. When *s*_*P*_ < 0.5, regardless of the initial consumer density, the production levels of the resources are insufficient to maintain any consumer population, and the system moves to the trivial equilibrium point.

If we take all *a*’s and *q*’s one tenth of the value in the first calculation, all stationary points have the same values, only the eigenvalues become complex, which implies oscillating graphs in the time plot (Fig. 4c) and in the phase plot (Fig. 4d) a trajectory initially heading for the trivial equilibrium and eventually spiralling into the stable coexistence point. Note that only the *BR*-plane is plotted, in the time plot it is clear the *P* and *R* are fully in phase.

### Two consumers and two resources

For two consumers *A* and *B* the two growth functions are just as for a single one

47 $$f_{A} (P,R) = f_{mA} \hbox{min} \left( {\frac{P}{{P + k_{PA} }},\frac{R}{{R + k_{RA} }}} \right),\quad f_{B} (P,R) = f_{mB} \hbox{min} \left( {\frac{P}{{P + k_{PB} }},\frac{R}{{R + k_{RB} }}} \right),$$

so again both resources need to have a certain minimal level. The zero growth isoclines for both consumers are the semi lines at

48 $$P_{A} = \frac{{k_{PA} m_{A} }}{{f_{mA} - m_{A} }},\;\;R_{A} = \frac{{k_{RA} m_{A} }}{{f_{mA} - m_{A} }},\;\;P_{B} = \frac{{k_{PB} m_{B} }}{{f_{mB} - m_{B} }},\;\;R_{B} = \frac{{k_{RB} m_{B} }}{{f_{mB} - m_{B} }},\;\;$$

provided we take *f*_*mA*_ > *m*_*A*_ and *f*_*mB*_ > *m*_*B*_. We choose parameters such that the isoclines do indeed intersect and that the intersection of two of the semi lines occurs at P* = *P*_*A*_ and R* = *R*_*B*_. Possible stationary points are the trivial one where the consumers are absent, one where only *A* is present, one with only *B*, and the coexistence point with both consumers. In all these points both resources are present.

Parameter values for the resources are *a*_*P*_ = 1, *a*_*R*_ = 1, *k*_*PA*_ = 0.9, *k*_*PB*_ = 0.7, *k*_*RA*_ = 0.8, and *k*_*RB*_ = 1, so *P*_*A*_ = 0.45, *P*_*B*_ = 0.35, *R*_*A*_ = 0.4, and *R*_*B*_ = 0.5. For the consumers the parameters are *m*_*A*_ = 1, *q*_*PA*_ = 1, *q*_*RA*_ = 0.8, *f*_*mA*_ = 3, and *m*_*B*_ = 1, *q*_*PB*_ = 0.8, *q*_*RB*_ = 1, *f*_*mB*_ = 3. In a second series we interchange the *q*’s. We study the behaviour of the system as a function of the stable resource levels *s*_*P*_ and *s*_*R*_.

**Fig. 5:**
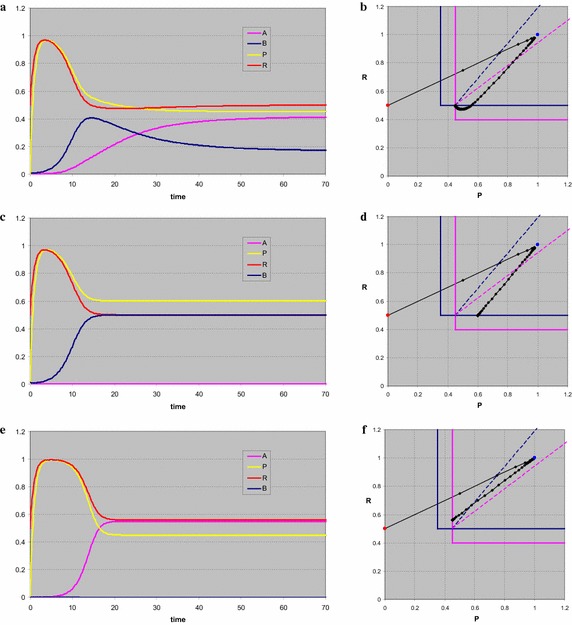
Stable coexistence of two consumers and two resources. The system first moves to the trivial equilibrium, the supply point, with only the resources, indicated by the *blue dot* in the phase plot (**b**). Next it develops to the point where only *B* is present, which (also) is a saddle point. Finally in **a**, **b** a stable coexistence of the consumers is reached at the intersection of the zero growth isoclines of *A* (*magenta*) and *B* (*blue*). The *thin dashed lines* indicate the consumption vectors of the two consumers. In **c**, **d** the initial density of species *A* is zero. Now the system behaves effectively as that of a single consumer and two resources. The resources quickly obtain their stable level, almost that is, since there still is a minute amount of consumer. Once consumption starts in earnest, both resources disappear in the ratio of the consumption vector of *B*, and an unstable state is reached, grace to the strict absence of *A*. In **e**, **f** the initial density of species *B* is set to zero. The system moves again to the trivial state, but next develops parallel to the consumption vector of *A* to the saddle point with only this species present. Since the initial density of *A* is quite low, the trivial state is approached very closely and in fact seems to be stable for a short time interval, until the density of *A* is sufficient to show it is a saddle point indeed

For *s*_*P*_ = 1, *s*_*R*_ = 1 (Fig. 5) the coexistence point of both consumers is a stable node, the other three equilibria are saddle points. All stationary densities are positive, so all stationary points are biologically relevant. For initial values *P*(0) = 0, *R*(0) = 0.5, *A*(0) = 0.001, *B*(0) = 0.01 the system first moves to the trivial equilibrium. Since that is a saddle point, by the time *B* is building up, the system moves into the direction of the *B*-point. Because *A* is present, that point too is unstable, and finally we end in the stable coexistence point with densities as specified by the theory: A* ≅ 0.41, B* ≅ 0.17, P* = 0.45, R* = 0.5. The time plot (Fig. 5a) shows the curves, the phase plot (Fig. 5b) shows the trajectory in the *PR*-plane, starting at the red dot. The blue dot is the position of the trivial equilibrium, the bold lines are the isoclines for *A* (magenta) and *B* (blue). The thin dashed lines represent the consumption vectors delimiting the region given by Eq. (35), with the colour referring to the consumer.

The same analysis but with *A*(0) = 0 (Fig. 5c, d) shows that the system now eventually moves to the saddle point of consumer *B*, which in the subspace in which we now move does establish a stable node at *B*_B_^*^ = 0.5, *P*_B_^*^ = 0.6 and *R*_B_^*^ = *R*_*B*_ = 0.5. Indeed three of the eigenvalues of this point are negative, all corresponding eigenvectors have zero for the *A*-component. Note that the latter part of the trajectory in the phase plot (Fig. 5d) moves parallel to the consumption vector of *B*, as for the case of one consumer and two resources.

If instead we set *B*(0) = 0 (Fig. 5e, f) the system moves even closer to the trivial equilibrium, because we start with just a tiny amount of *A*. Once this consumer density starts growing exponentially, the system rapidly moves towards the saddle point of *A*, with *A*_A_^*^ = 0.55, *P*_A_^*^ = *P*_*A*_ = 0.45 and *R*_A_^*^ = 0.56. Note that now the latter part of the trajectory (Fig. 5f) is parallel to the consumption vector of *A*.

**Fig. 6:**
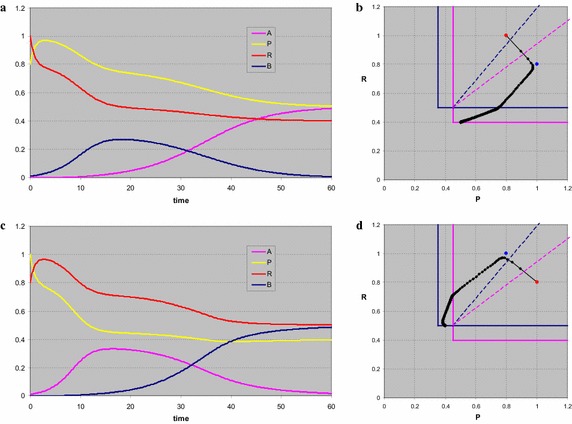
When the supply point (*blue dot*) for the resources lies outside the wedge as defined by the consumption vectors, there cannot be coexistence of both consumers. In both cases the system first develops to the trivial point. In **a**, **b** next the system moves towards the point with only *B*. Both are saddles and eventually the stable point with only *A* is reached. Note that between the two saddles the trajectory in (**b**) is parallel to the consumption vector of *B*. When the supply point is above the wedge (**d**), the roles of the two consumers are interchanged. After heading for the trivial state the trajectory moves parallel to the consumption vector of *A* to the saddle point with only *A*, before ending in the stable point with only the *B* present. Note that time plots **a**, **c** are almost identical, up to the role switch between both consumers and resources

When the supply point for the resources lies outside the wedge as defined by the consumption vectors, there cannot be coexistence of both consumers. For *s*_*P*_ = 1, *s*_*R*_ = 0.8 (Fig. 6a, b) the coexistence point is not biologically relevant (at least one of the consumer densities is negative), the *A*-point is a stable node, the other two equilibria are saddle points. If we start in *P*(0) = 0.8, *R*(0) = 1, *A*(0) = 0.0001, *B*(0) = 0.01, the system moves to the trivial point, then to the *B*-point, and finally the *A*-point at with *A*_A_^*^ = 0.5, *P*_A_^*^ = 0.5 and *R*_A_^*^ = *R*_*A*_ = 0.4. For *s*_*P*_ = 0.8, *s*_*R*_ = 1 (Fig. 6c, d) the situation is reversed and we end up in *B*_B_^*^ = 0.5, *P*_B_^*^ = 0.395 and *R*_B_^*^ = *R*_*B*_ = 0.5. Note that the two time plots are almost identical, apart from the switch between the consumers and the resources. This is due to the similarity in the other parameter values and the specific choice of the initial points.

**Fig. 7:**
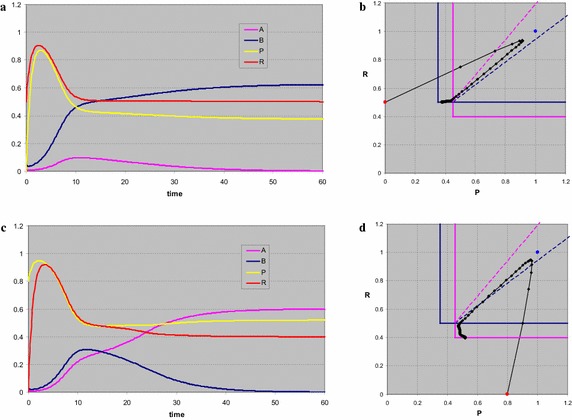
For stable coexistence of the consumers not only the supply of the resource must suffice, but also each consumer should consume resources in a ratio that favours the competitor. If such is not the case, the coexistence point is a saddle. With the supply point inside the wedge there are two stable states, each with only one consumer present. Which consumer survives depends entirely on the initial state. In **a**, **b** after moving to the trivial state and the coexistence point only the *B* survives. For a different initial state the system develops to a situation in which *A* eventually prevails. The trajectories as shown seem to coincide partially. This occurs because only the phase plot projected onto the *PR*-plane is given. In the full phase space, including the state variables *A* and *B*, the basins of the two stable equilibria do not overlap

A quite different situation is found when we take *q*_*PA*_ = 0.8, *q*_*RA*_ = 1, *q*_*PB*_ = 1, and *q*_*RB*_ = 0.8 (Fig. 7). For *s*_*P*_ = 1, *s*_*R*_ = 1 the coexistence point and the trivial equilibrium are saddle points, the other two equilibria are stable nodes. All stationary densities are positive, so all stationary points are biologically relevant. For initial values *P*(0) = 0,  *R*(0) = 0.5,  *A*(0) = 0.01,  *B*(0) = 0.05 the systems travels via the trivial and coexistence point to the stable *B*-point (Fig. 7a, b). If we start in *P*(0) = 0.8, *R*(0) = 0, *A*(0) = 0.01, *B*(0) = 0.03, a similar detour brings us to the stable *A*-point (Fig. 7c, d). The two stable points each have their own basin of attraction, the choice of the starting point completely determines where the system will end up. Note that it looks as if the two trajectories in Fig. 7b, d intersect. In fact they are fully separated, any apparent intersection occurs because the projection of the orbit upon the *PR*-plane is plotted.

**Fig. 8:**
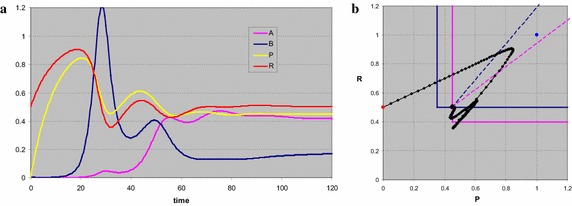
Time (**a**) and phase plot (**b**) for a system in which the coexistence point of the two consumers is a stable vortex. First the resources build up, and the trivial state is approached. When *B* builds up the resources are consumed in a fixed proportion, and the state with only *B* is approached. When finally *A* builds up the system oscillates into the coexistence point at the intersection of the isoclines

The same system as the first in this series, but now with all the *a*’s and *q*’s divided by ten, again shows the oscillating behaviour around exactly the same stationary points (Fig. 8). If the time scales in the system are sufficiently different, the eigenvalues of the Jacobi matrices in the stationary points can be complex. In the phase plot (Fig. 8b) the projection of the trajectory on the *PR* plane does not show the spiralling behaviour, as corroborated by the observation that in the time plot (Fig. 8a) the resource densities are only slightly out of phase.

Once again the importance of the saddle points is exemplified by the majority of the above results. In all cases the system first moves along the stable direction of a saddle point towards it, only to move away after it has switched to the unstable direction. Depending on the detailed values of the parameters this may take arbitrarily long. For each dynamical system there are trajectories that travel about its phase space on a detour around the saddle points before finally ending up in a stable equilibrium.

## Conclusions and discussion

The conclusions from these mathematical analyses and simulations are quite generic. For all systems investigated the stationary points are either saddle points or stable nodes or vortices. Moreover there always is at least one stable equilibrium. For the resource that is not surprising, that feature is introduced explicitly in the model. For the consumers it is the result of the interaction. If the available amount of food is sufficient to overcome the inherent mortality of the consumer, it will increase in numbers. That is quite trivial and introduced manually into the model too. What is not so trivial is that an increase in consumer density does not necessarily lead to such a decrease of the food supply that it falls below the required abundance, setting off a chain of events that eventually leads to extinction of the consumer or an infinite repetition of events. A scenario like this is feasible, but in the current model it does not happen. Instead the system converges to a stable coexistence point, like in Fig. 2d or Fig. 4d where the trajectory spirals into the stable vortex.

Another generic feature of the models is that if there are biologically relevant stationary points (either internal or boundary equilibria for the consumers) at least one of these is a stable point. Again this may not be very surprising, but there are also physically relevant stationary points that are unstable. In such cases we always have saddle points, there are no unstable nodes or vortices. That the saddle points are important is shown in the numerical section. In all cases where we start with an almost empty system it first develops towards a saddle point, and lingers there for a considerable time before eventually moving away from it, heading for a stable equilibrium, or yet another saddle point. Of course the actual dynamics depend on the initial condition, but the main message is that whenever the system comes near a saddle point, it may stay there for any length of time, depending on actually how unstable the local equilibrium is. Non-linear systems are known to possess also other type of equilibria, such as (quasi)periodic, homoclinic or heteroclinic orbits or strange attractors in chaotic systems. The systems as discussed here have none of these, though the orbit along the saddle points may be seen as a sort of precursor of a heteroclinic orbit, with the difference that it ends in a stable equilibrium instead of closing upon itself. Saddle points are abundant in complex systems, and they have a significant impact on the dynamics of such systems. The present ones are not very complex but they are no exception. There is nothing exotic about saddle points, they exist for any choice of parameters, the only issue is whether or not the system comes near them. If the instability of a stationary point is the result of a change in one or more of the model parameters, reflecting a change in system properties, such a system may be arbitrary close to a saddle point. The systems we study also are fully deterministic, there is no stochasticity involved, and all parameters are fully constant. In practice it may not be possible to distinguish whether an observed ecosystem is close to a stable equilibrium, or to a saddle point. In fact, with ever changing external factors and the many degrees of freedom in real systems, the concept of a stable equilibrium may not be very relevant, and a spectrum of eigenvalues that indicates how rapidly a given situation may destabilise, is much more useful. It is surprising that such simple equations as the ones discussed here already show the onset of complicated system behaviour, like in Fig. 3b where the unstable coexistence between consumer *B* and the resource pertains for quite a while before the more successful invader *A* takes over. Coexistence of two consumers depending on just a single resource is possible within the Tilman model, as is survival of a population which is being invaded by a more efficient consumer, be it for a finite period. Eventually the model system does develop towards a stable equilibrium, the relevant question is about the rate at which the crossover occurs.

The generic conclusions, by definition, are rather qualitative. Mathematics, however, is largely geared to produce quantitative results, provided of course we give specific input to the model. If we combine the generic result of Eq. (8) with the one (40) for the specific growth function as studied, we obtain the coexistence point of a single consumer and a single resource as a function of the model parameters

49 $$B{\kern 1pt} ^* = \frac{{a_{R} (s_{R} - R{\kern 1pt} ^*)}}{{q_{RB} m_{B} }},\;\;R{\kern 1pt} ^* = \frac{{k_{RB} m_{B} }}{{f_{mB} - m_{B} }}.$$

In fact we know much more, from the eigenvalues and eigenvectors of the Jacobi matrix we know exactly how and how fast this stable equilibrium is approached if the parameters are such that 0 < R* < *s*_*R*_. Depending on the available experimental data we can use this to estimate these parameters and decide upon the goodness of fit whether the model provides a satisfactory description, or whether it should be modified. Alternatively we can accept the model and investigate for instance how the equilibrium behaves as a function of the half saturation constant *k*_*RB*_, keeping all other parameters constant. The maximal value of the consumer density mathematically is reached for a zero value of this parameter, and the resource density is zero too. Ecologically this seems a pathology, but as explained in the introduction of the model we could have expected this. By virtue of the autonomous dynamics of the resource within the model the resource production is maximized at zero resource density. That it really is a pathology is clear when we realise that for *k*_*RB*_ = 0 the growth function (42) of the consumer is constant, independent of the resource density. This violates the condition that the growth function should be zero for zero resource density. For any finite but (very) small value of *k*_*RB*_ the situation in fact is normal. The equilibrium resource density increases linearly with *k*_*RB*_, and the consumer density decreases linearly, until it becomes zero when R* = *s*_*R*_. The ecological rationale is that if the consumer really needs only a small amount of resource to be present to overcome its inherent mortality, it can take maximal profit from it. The growth function and the conversion factor reflect the details of the foraging for and digestion of the resource, but the theory does not give us the exact relation. Additional modelling would be needed.

Slightly more complicated is the case of one consumer and two food sources. As stated, it seems like this case allows the consumer to opt for a trade-off between the two resources, and choose whichever can be obtained most easily. The model says differently. Combining (22) and (46b) we find the coexistence point

50 $$B{\kern 1pt} ^* = \frac{{a_{R} (s_{R} - R{\kern 1pt} ^*)}}{{q_{RB} m_{B} }},\;\;P{\kern 1pt} ^* = s_{P} - \frac{{a_{R} q_{PB} }}{{a_{P} q_{RB} }}(s_{R} - R{\kern 1pt} ^*),\;\;R{\kern 1pt} ^* = \frac{{k_{RB} m_{B} }}{{f_{mB} - m_{B} }} .$$

If 0 < R* < *s*_*R*_, and the same for the other resource, this is a stable equilibrium. Since the stable resource densities are fixed, so are the resource production rates, and the two consumption rates that compensate these. The consumer has no choice, the ratio of consumption of the two different resources is determined by that of the two conversion factors *q*. These conversion factors were introduced into the model to allow for a difference in increase in consumer density, in terms of individuals per unit surface or volume, and corresponding decrease in resource density, in similar units. In the setting we have used the *q*’s are inverse yield factors. If the densities are expressed as biomass instead of individual densities, the *q*’s might be understood as determining the efficiency of the process, but again we must be careful. If the resource is for instance salt, and a second resource such as water because of its ample availability is left out of the model, a small intake of salt could lead to a huge increase in biomass. It helps in such a case to use the dry weight for defining biomass, but that only reduces the discrepancy. For essential resources, as we have used in the numerical calculations, some authors (Dybzinski and Tilman 2012; Alstad 2007) assume the ratio of the resource uptakes to be the same as that of the minimally required resource densities to compensate for the mortality

51 $$P_{B} = \frac{{k_{PB} m_{B} }}{{f_{mB} - m_{B} }},\;\;R_{B} = \frac{{k_{RB} m_{B} }}{{f_{mB} - m_{B} }},\;\;\frac{{q_{PB} }}{{q_{RB} }} = \frac{{P_{B} }}{{R_{B} }} = \frac{{k_{PB} }}{{k_{RB} }}.$$

It helps of course to reduce the number of model parameters, but in fact we are dealing with two quite different things. As we have seen before, a very low resource density leads to a very large resource production, allowing for a very large resource uptake. Even if we ignore the aspects of the conversion factor as we have just mentioned, it is wrong to assume a proportionality between the resource uptake and density. Of course it is possible to modify the model to allow for a choice on the part of the consumer, by introducing just one of the two and having the ratio determined by an optimisation procedure of a separate model for the trade-off between the resources, yet to be specified. Instead of reducing the number of parameters, this will likely lead to an increase of the number of parameters in an extended model with optimal foraging.

The case of two consumers and two resources proves to be a very complex system, given the simplicity of the equations. This should be no surprise. Indeed, systems with as little as three coupled non-linear ordinary differential equations can show chaotic behaviour with strange attractors. So four such equations could have given even more fireworks, but apparently these don’t. Actually it has been shown (Huisman and Weissing 1999) that three consumers with three resources can show chaotic behaviour, which might suggest that the equations for the resources don’t add to the complexity. In fact chaotic 3D systems can be of the Lotka-Volterra direct competition type, and the whole purpose of the Tilman model is to provide a more indirect specification of the consumer interactions, through the resources. A Lotka-Volterra-like 2D consumer system can show similar behaviour as the 4D Tilman system. Whether the Tilman models really have the advantage of connecting more directly to ecological systems than equivalent Lotka-Volterra system cannot be answered on the base of this investigation. We did argue that the additional parameters introduced by the extra equations for the resource still take on effective values when compared to experiments.

Although the equilibria and their stability are explained in the majority of ecological text books, the transient dynamics and how different time scales are involved in these are not. With this paper we contribute to the understanding of the dynamics of competing organisms in a non-equilibrium context. Due to changing environments more often than not systems are not in equilibrium, and the transitional dynamics is more relevant than the equilibrium one.

## List of symbols

For each symbol used in the text also its physical dimension is given in brackets. Here [L] is the dimension of a length, and [T] the dimension of time. Numbers are taken to be dimensionless, indicated as [0].

*A*: Consumer density [L^−3^]
*a*_*j*_: Growth factor for resource *j* (either *P* or *R*) [T^−1^]
*B*: Consumer density [L^−3^]
*c*_*n*_: Coefficient of the characteristic polynomial (of the *n* × *n* Jacobi matrix) [T^−*n*^]
*F*: Flow [L^3^T^−1^]
*f*_*i*_(): Growth function for consumer *i* (either *A* or *B*) [T^−1^]
*f*_*mi*_: Maximum of the growth function for consumer *i* [T^−1^]
*J*(): Jacobi matrix [T^−1^]
*k*_*ji*_: Half saturation constant of resource *j* in growth function of consumer *i* [L^−3^]
λ: Eigenvalue (of the Jacobi matrix) [T^−1^]
*m*_*i*_: Mortality rate of consumer *i* [T^−1^]
*N*_*i*_: Density of consumer *i* [L^−3^]
*P*: Resource density [L^−3^]
*q*_*ji*_: Conversion factor from resource *j* to consumer *i* [0]
*r*: Resource density [L^−3^]
*R*: Resource density [L^−3^]
*R*_*j*_: Density of resource *j* [L^−3^]
*s*_*j*_: Stable level for resource *j* [L^−3^]
*t*: Time [T]
*V*: Volume [L^3^]

## Appendix A: Dynamical systems

Here we recapitulate some basic mathematics of dynamical systems. This appendix may also serve as a glossary for the mathematical terminology we use in this paper. For a full overview of the mathematics involved we refer the reader to the general literature on the subject, such as (Edelstein-Keshet 2005).

A *dynamical system* in the context as we use it here is a set of *differential equations* describing the time development of a number of *variables*. In this case the variables are the consumer and resource densities. Because of the interaction between the consumers and the resources the time development of their densities depends on in principle all density values. For two such variables, one consumer and one resource, the dynamical system is

52 $$\left\{ \begin{array}{l} \frac{dB(t)}{dt} = f(B(t),R(t))\\ \frac{dR(t)}{dt} = g(B(t),R(t))\\ \end{array} \right.$$

The functions on the right hand side specify exactly how the rate of change of the variables depends on the values of the variables. Given the values of those variables, their time derivative is fully specified. The values of the variables define what is called the *state* of the system and the variables are called *state variables*. In most applications the state variables must have a positive value to be biologically relevant. If a variable in the mathematical model has a negative value, it is called *unphysical*, or not biologically relevant. The functions on the right hand side also contain several constants, such as the mortality of the consumer and the supply of the resource. These constants are called *parameters*. For different parameter values the same dynamical system may show different behaviour. We call this the *dynamics* of the system.

The behaviour of the system can be visualized graphically by making a *time plot* of the graphs of the variables as a function of time. The state of a system with *n* variables can be represented by a point in an *n*-dimensional real space, the *phase space*. The consecutive states establish a continuous curve in phase space, called a *trajectory*. For a system with just two state variables an *xy*-plot, with one variable along the horizontal and the other along the vertical axis, depicts the full phase space. For systems with more variables generally only a projection of the phase space is given in a *phase plot*.

An important dynamical feature are the *stationary states* of a dynamical system. For a stationary state all variables are constant with time, all time derivatives are zero. Alternative terms are *stationary point* or *equilibrium*. Stationary states are found by mathematically solving a system of algebraical equations

53 $$\left\{ \begin{aligned} f(B,R) = 0 \hfill \\ g(B,R) = 0 \hfill \\ \end{aligned} \right.$$

with one equation for each (constant) state variable. In general this system, like the dynamical system, is non-linear. That implies it may have several solutions, which need not all be biologically relevant. For each of these equilibria the stability can be assessed by making a linear approximation to the non-linear dynamical system, about the stationary point. If

54 $$\left\{ \begin{aligned} \delta (t) = B(t) - B_{0} \hfill \\ \varepsilon (t) = R(t) - R_{0} \hfill \\ \end{aligned} \right.$$

are small *perturbations* about the stationary state *B* = *B*_0_, *R* = *R*_0_, these approximately satisfy

55 $$\left\{ \begin{aligned} \frac{d\delta }{dt} = \frac{\partial f(B,R)}{\partial B}\delta (t) + \frac{\partial f(B,R)}{\partial R}\varepsilon (t) \hfill \\ \frac{d\varepsilon }{dt} = \frac{\partial g(B,R)}{\partial B}\delta (t) + \frac{\partial g(B,R)}{\partial R}\varepsilon (t) \hfill \\ \end{aligned} \right..$$

This is a linear system of differential equations, which can be written in matrix–vector format

56 $$\left( {\begin{array}{*{20}c} {\delta^{\prime}(t)} \\ {\varepsilon^{\prime}(t)} \\ \end{array} } \right) = \left( {\begin{array}{*{20}c} {\frac{\partial f(B,R)}{\partial B}} & {\frac{\partial f(B,R)}{\partial R}} \\ {\frac{\partial g(B,R)}{\partial B}} & {\frac{\partial g(B,R)}{\partial R}} \\ \end{array} } \right)\left( {\begin{array}{*{20}c} {\delta (t)} \\ {\varepsilon (t)} \\ \end{array} } \right) \equiv J(B,R)\left( {\begin{array}{*{20}c} {\delta (t)} \\ {\varepsilon (t)} \\ \end{array} } \right).$$

The partial derivatives in the *Jacobi matrix**J*(*B*, *R*) are evaluated at the individual stationary point investigated. Each point may have a different matrix. The equilibrium is stable if for any initial perturbation in the limit of large *t* it approaches the equilibrium, and unstable otherwise. To establish stability properties it normally suffices to calculate the eigenvalues of the Jacobi matrix. The eigenvalues λ have the dimension of 1/*t*, the inverse of an eigenvalue defines a *time scale*. A system with any number of state variables is stable if the real parts of all eigenvalues of the Jacobi matrix are negative, and unstable otherwise. More specifically if all eigenvalues are real and negative, the equilibrium is a *stable node*. In the time plot all graphs relax exponentially to that state, with a relaxation rate corresponding to the largest eigenvalue (less negative one). If eigenvalues are complex with all negative real parts, the equilibrium is a *stable vortex*. The time plot shows oscillatory relaxation, in a phase plot the trajectory spirals into the equilibrium point. If all eigenvalues are positive, or have a positive real part, we speak of an *unstable node* or *unstable vortex*. The graphical behaviour is as for stable points, backwards in time. If some eigenvalues are positive and some negative we speak of a *saddle point*. Near a saddle point the system is stable in some directions, but unstable in others. Eventually it will move in the unstable directions, and hence is unstable, but in practice this may take a long time, depending on the time scales involved.

## Appendix B: Mathematical details of the stability analysis

### Single species consuming a single resource

The Jacobi matrix for system (2a, 2b) is given by

57 $$J(B,R) = \left( {\begin{array}{*{20}c} {f_{B} (R) - m_{B} } & {B\frac{{df_{B} }}{dR}} \\ { - q_{RB} f_{B} (R)} & { - a_{R} - q_{RB} B\frac{{df_{B} }}{dR}} \\ \end{array} } \right).$$

For the trivial equilibrium (3) we find

58 $$J(0,s_{R} ) = \left( {\begin{array}{*{20}c} {f(s_{R} ) - m_{B} } & 0 \\ { - q_{RB} f_{B} (s_{R} )} & { - a_{R} } \\ \end{array} } \right),$$

with eigenvalues *λ*_1_ = *f*_*B*_(*s*_*R*_) − *m*_*B*_ and *λ*_2_ = −*a*_*R*_. For any biologically relevant situation the parameter *a*_*R*_ is positive, so one of the eigenvalues is always negative. If the mortality is larger than *f*_*B*_(*s*_*R*_), also the second eigenvalue is negative, and the stationary point (0, *s*_*R*_) is a stable node. If the mortality is smaller than *f*_*B*_(*s*_*R*_), the first eigenvalue is positive, the stationary point is a saddle point, which is unstable. The Jacobi matrix for the coexistence point (8) is

59 $$J(B{\kern 1pt} ^*,R{\kern 1pt} ^*) = \left( {\begin{array}{*{20}c} 0 & {B{\kern 1pt} ^*\left. {\frac{{df_{B} }}{dR}} \right|_{R = R{\kern 1pt} ^*} } \\ { - q_{RB} m_{B} } & { - a_{R} - q_{RB} B{\kern 1pt} ^*\left. {\frac{{df_{B} }}{dR}} \right|_{R = R{\kern 1pt} ^*} } \\ \end{array} } \right).$$

The sum of the eigenvalues (the trace of the matrix) is negative and their product (the determinant of the matrix) is positive, so both eigenvalues have a negative real part, and the equilibrium is stable.

If *f*_*B*_(∞) < *m*_*B*_, the only stationary point is the stable trivial equilibrium. Because the growth rate of *B* is smaller than its mortality for any value of *R*, the net relative growth rate of *B* will always be negative, more negative than *f*_*B*_(∞) − *m*_*B*_, so *B* will die out. Once it does, *R* will go to its stable level *s*_*R*_, the trivial equilibrium is a global attractor. If *f*_*B*_(∞) > *m*_*B*_, and *R** > *s*_*R*_ the equilibrium density for the consumer in the coexistence point is negative, which is unphysical. Moreover this point can never be reached from any biologically relevant initial situation, since according to (2a) *dB*/*dt* = 0 for *B* = 0, regardless of *R*, while according to (2b) *dR*/*dt* > 0 for *R* = 0, regardless of *B*. The fact that *f*_*B*_(*R*) is an increasing function of *R* implies that in this case *f*_*B*_(*s*_*R*_) < *m*_*B*_, so the trivial stationary point is stable. The global behaviour can be understood by looking at *R* first. If $R(0) \ge R{\kern 1pt} ^*$ the amount of available resource is initially sufficient to have the population *B* grow. However, since the replenishing level is below *R*(0), *dR*/*dt* < 0, so *R* will drop until it is no longer sufficient to sustain growth and also *B* will drop, and will continue to do so. Eventually *B* will drop to zero and *R* will reach its stable level *s*_*R*_. Again the trivial equilibrium is globally stable.

### Two species competing for a single resource

The Jacobi matrix for the system (11a, 11b, 11c) is

60 $$J(A,B,R) = \left( {\begin{array}{*{20}c} {f_{A} (R) - m_{A} } & 0 & {A\frac{{df_{A} }}{{dR_{{}} }}} \\ 0 & {f_{B} (R) - m_{B} } & {B\frac{{df_{B} }}{{dR_{{}} }}} \\ { - q_{RA} f_{A} (R)} & { - q_{RB} f_{B} (R)} & { - a_{R} - q_{RA} A\frac{{df_{A} }}{dR} - q_{RB} B\frac{{df_{B} }}{dR}} \\ \end{array} } \right).$$

We take *R*_*A*_^*^ < *R*_*B*_^*^. If *s*_*R*_ < *R*_*A*_^*^ both (14) and (16) are unphysical. For the trivial equilibrium

61 $$J(0,0,S) = \left( {\begin{array}{ccc} f_{A} (s_{R} ) - m_{A} & 0 & 0 \\ 0 & f_{B} (s_{R} ) - m_{B} & 0 \\ - q_{RA} f_{A} (s_{R} ) & - q_{RB} f_{B} (s_{R} ) & - a_{R} \\ \end{array} } \right).$$

The three eigenvalues are the three diagonal elements of the Jacobi matrix. All three are negative so the trivial equilibrium is a stable node. For any positive initial value for *A* and/or *B*, and a *R*(0) > *s*_*R*_, the resource will see a net depletion, as all terms in (11c) are negative. *A* and *B* may grow, depending on the actual value of *R*(0), but eventually *R* will drop to the stable level *s*_*R*_. If at that time *A* and *B* happen to be zero, we have reached the equilibrium point, if not, they will both decrease because their mortality exceeds the level of their growth function. Also *R* will drop, because both consumers are still eating it away. Eventually both species will become extinct in the sample area, after which the resource is replenished to its stable level. The local stable node is a global attractor in the physical domain.

When *R*_*A*_^*^ < *s*_*R*_ < *R*_*B*_^*^, the Jacobi matrix for the trivial equilibrium (61) has two negative eigenvalues and one positive one, so it is a saddle point. The Jacobi matrix for equilibrium (14) is

62 $$J(A^*,0,R_{A}^{*} ) = \left( {\begin{array}{*{20}c} 0 & 0 & {A ^*\left. {\frac{{df_{A} }}{dR}} \right|_{{R = R_{A}^{*} }} } \\ 0 & {f_{B} (R_{A}^{*} ) - m_{B} } & 0 \\ { - q_{RA} m_{A} } & { - q_{RB} f_{B} (R_{A}^{*} )} & { - a_{R} - q_{RA} A^ *\left. {\frac{{df_{A} }}{dR}} \right|_{{R = R_{A}^{*} }} } \\ \end{array} } \right).$$

One of the eigenvalues is *f*_*B*_(*R*_*A*_^*^) − *m*_*B*_, which is a negative number. The sum of the other two eigenvalues (*J*_3,3_) is negative, their product (−*J*_3,1_*J*_1,3_) is positive, so they both have a negative real part. The equilibrium is a stable node or a stable vortex.

When *s*_*R*_ < *R*_*A*_^*^ < *R*_*B*_^*^, the trivial equilibrium has two positive and one negative eigenvalue, so it is saddle. Equilibrium (14) still has the Jacobi matrix of type (62), so it still is a stable node or a stable vortex. The Jacobi matrix for (16) is

63 $$J(0,B{\kern 1pt} ^*,R_{B}^{*} ) = \left( {\begin{array}{*{20}c} {f_{A} (R_{B}^{*} ) - m_{A} } & 0 & 0 \\ 0 & 0 & {B{\kern 1pt} ^*\left. {\frac{{df_{B} }}{dR}} \right|_{{R = R_{B}^{*} }} } \\ { - q_{RA} f_{A} (R_{B}^{*} )} & { - q_{RB} m_{B} } & { - a_{R} - q_{{_{R} B}} B{\kern 1pt} ^*\left. {\frac{{df_{B} }}{dR}} \right|_{{R = R_{B}^{*} }} } \\ \end{array} } \right).$$

One of the eigenvalues is *f*_*A*_(*R*_*B*_^*^) − *m*_*A*_, which is a positive number. The sum of the other two eigenvalues (*J*_3,3_)is negative, their product (−*J*_2,1_*J*_1,2_) is positive, so they both have a negative real part. The equilibrium is a saddle point.

### A single species consuming two different resources

For the system of a single species depending on two different resources (17a, 17b, 17c), the Jacobi matrix is

64 $$J(B,P,R) = \left( {\begin{array}{*{20}c} {f_{B} (P,R) - m_{B} } & {B\frac{{\partial f_{B} }}{\partial P}} & {B\frac{{\partial f_{B} }}{\partial R}} \\ { - q_{PB} f_{B} (P,R)} & { - a_{P} - q_{PB} B\frac{{\partial f_{B} }}{\partial P}} & { - q_{PB} B\frac{{\partial f_{B} }}{\partial R}} \\ { - q_{RB} f_{B} (P,R)} & { - q_{RB} B\frac{{\partial f_{B} }}{\partial P}} & { - a_{R} - q_{RB} B\frac{{\partial f_{B} }}{\partial R}} \\ \end{array} } \right).$$

The Jacobi matrix in the trivial equilibrium is

65 $$J(0,s_{P} ,s_{R} ) = \left( {\begin{array}{*{20}c} {f_{B} (s_{P} ,s_{R} ) - m_{B} } & 0 & 0 \\ { - q_{PB} f_{B} (s_{P} ,s_{R} )} & { - a_{P} } & 0 \\ { - q_{RB} f_{B} (s_{P} ,s_{R} )} & 0 & { - a_{R} } \\ \end{array} } \right).$$

The diagonal elements are the eigenvalues. The last two are negative, the first is negative if the coexistence point is unphysical and positive otherwise. If there is a biologically relevant coexistence point the trivial equilibrium is a saddle point, otherwise it is a stable node.

The Jacobi matrix in the coexistence point is

66 $$J(B{\kern 1pt} ^*,P{\kern 1pt} ^*,R{\kern 1pt} ^*) = \left( {\begin{array}{*{20}c} 0 & {B{\kern 1pt} ^*\frac{{\partial f_{B} }}{\partial P}} & {B{\kern 1pt} ^*\frac{{\partial f_{B} }}{\partial R}} \\ { - q_{PB} m_{B} } & { - a_{P} - q_{PB} B{\kern 1pt} ^*\frac{{\partial f_{B} }}{\partial P}} & { - q_{PB} B{\kern 1pt} ^*\frac{{\partial f_{B} }}{\partial R}} \\ { - q_{RB} m_{B} } & { - q_{RB} B{\kern 1pt} ^*\frac{{\partial f_{B} }}{\partial P}} & { - a_{R} - q_{RB} B{\kern 1pt} ^*\frac{{\partial f_{B} }}{\partial R}} \\ \end{array} } \right),$$

with the gradients of the growth function evaluated at the intersection point. All parameters and variables have positive values, so the trace of the matrix is negative, and also the determinant is negative. Moreover, the characteristic polynomial *λ*^3^ + *c*_1_*λ*^2^ + *c*_2_*λ* + *c*_3_ has all positive coefficients, with *c*_1_ minus the trace and *c*_3_ minus the determinant, and also satisfies the Routh-Hurwitz criterion *c*_1_*c*_2_ > *c*_3_ (see Edelstein-Keshet 2005). This implies all eigenvalues have a negative real part, the stationary point is stable if it is physical.

### Two species and two resources

The general Jacobi matrix of the system of equations (23a, 23b, 23c, 23d) is

67 $$J = \left( {\begin{array}{*{20}c} {f_{A} - m_{A} } & 0 & {A\frac{{\partial f_{A} }}{\partial P}} & {A\frac{{\partial f_{A} }}{{\partial R_{{}} }}} \\ 0 & {f_{B} - m_{B} } & {B\frac{{\partial f_{B} }}{\partial P}} & {B\frac{{\partial f_{B} }}{{\partial R_{{}} }}} \\ { - q_{PA} f_{A} } & { - q_{PB} f_{B} } & { - a_{P} - {\kern 1pt} q_{PA} A\frac{{\partial f_{A} }}{\partial P} - {\kern 1pt} q_{PB} B\frac{{\partial f_{B} }}{\partial P}} & { - q_{PA} A\frac{{\partial f_{A} }}{\partial R} - q_{PB} B\frac{{\partial f_{B} }}{{\partial R_{{}} }}} \\ { - q_{RA} f_{A} } & { - q_{RB} f_{B} } & { - q_{RA} A\frac{{\partial f_{A} }}{\partial P} - q_{RB} B\frac{{\partial f_{B} }}{\partial P}} & { - a_{R} - {\kern 1pt} q_{RA} A\frac{{\partial f_{A} }}{\partial R} - {\kern 1pt} q_{RB} B\frac{{\partial f_{B} }}{\partial R}} \\ \end{array} } \right).$$

The Jacobi matrix at the trivial equilibrium point is

68 $$J(0,0,s_{P} ,s_{R} ) = \left( {\begin{array}{*{20}c} {f_{A} (s_{P} ,s_{R} ) - m_{A} } & 0 & 0 & 0 \\ 0 & {f_{B} (s_{P} ,s_{R} ) - m_{B} } & 0 & 0 \\ { - q_{PA} f_{A} (s_{P} ,s_{R} )} & { - q_{PB} f_{B} (s_{P} ,s_{R} )} & { - a_{P} } & 0 \\ { - q_{RA} f_{A} (s_{P} ,s_{R} )} & { - q_{RB} f_{B} (s_{P} ,s_{R} )} & 0 & { - a_{R} } \\ \end{array} } \right).$$

Again the eigenvalues are the diagonal elements. If the stable level of the resources is sufficient to overcome the mortality of at least one of the consumers, it is a saddle point, otherwise it is a stable node. The Jacobi matrix for the *A*-point is

69 $$J(A^{\prime},0,P_{A}^{*} ,R_{A}^{*} ) = \left( {\begin{array}{*{20}c} 0 & 0 & {A^{\prime}\frac{{\partial f_{A} }}{\partial P}} & {A^{\prime}\frac{{\partial f_{A} }}{\partial R}} \\ 0 & {f_{B} (P_{A}^{*} ,R_{A}^{*} ) - m_{B} } & 0 & 0 \\ { - q_{PA} m_{A} } & { - q_{PB} f_{B} (P_{A}^{*} ,R_{A}^{*} )} & { - a_{P} - q_{PA} A^{\prime}\frac{{\partial f_{A} }}{\partial P}} & { - q_{PA} A^{\prime}\frac{{\partial f_{A} }}{{\partial R_{{}} }}} \\ { - q_{RA} m_{A} } & { - q_{RB} f_{B} (P_{A}^{*} ,R_{A}^{*} )} & { - q_{RA} A^{\prime}\frac{{\partial f_{A} }}{\partial P}} & { - a_{R} - q_{RA} A^{\prime}\frac{{\partial f_{A} }}{\partial R}} \\ \end{array} } \right)$$

with derivatives at *P* = *P*_*A*_^*^, *R* = *R*_*A*_^*^. One eigenvalue is *λ* = *f*_*B*_(*P*_*A*_^*^, *R*_*A*_^*^) − *m*_*B*_, the others are the same as for one consumer and two resources, not very surprising. If the growth function for consumer *B* in the *A*-point exceed the mortality *m*_*B*_, the *A*-point is a saddle, if not it is a stable node or vortex. Similar arguments apply for the *B*-point. Finally the coexistence point has Jacobi matrix

70 $$J{\kern 1pt} ^* = \left( {\begin{array}{*{20}c} 0 & 0 & {A^ *\frac{{\partial f_{A} }}{\partial P}} & {A^*\frac{{\partial f_{A} }}{{\partial R_{{}} }}} \\ 0 & 0 & {B{\kern 1pt} ^*\frac{{\partial f_{B} }}{\partial P}} & {B{\kern 1pt} ^*\frac{{\partial f_{B} }}{{\partial R_{{}} }}} \\ { - q_{PA} m_{A} } & { - q_{PB} m_{B} } & { - a_{P} - {\kern 1pt} q_{PA} A^*\frac{{\partial f_{A} }}{\partial P} - {\kern 1pt} q_{PB} B{\kern 1pt} ^*\frac{{\partial f_{B} }}{\partial P}} & { - q_{PA} A^ *\frac{{\partial f_{A} }}{\partial R} - q_{PB} B{\kern 1pt} ^*\frac{{\partial f_{B} }}{{\partial R_{{}} }}} \\ { - q_{RA} m_{A} } & { - q_{RB} m_{B} } & { - q_{RA} A^ *\frac{{\partial f_{A} }}{\partial P} - q_{RB} B{\kern 1pt} ^*\frac{{\partial f_{B} }}{\partial P}} & { - a_{R} - {\kern 1pt} q_{RA} A^*\frac{{\partial f_{A} }}{\partial R} - {\kern 1pt} q_{RB} B{\kern 1pt} ^*\frac{{\partial f_{B} }}{\partial R}} \\ \end{array} } \right),$$

with $J{\kern 1pt} ^* = J(A^*,{\kern 1pt} B{\kern 1pt} ^*,{\kern 1pt} P{\kern 1pt} ^*,{\kern 1pt} R{\kern 1pt} ^*)$ and derivatives taken at $P = P{\kern 1pt} ^*,\;\;R = R{\kern 1pt} ^*$. The determinant is

71 $$\det (J{\kern 1pt} ^*) = m_{A} A{\kern 1pt} ^*m_{B} B{\kern 1pt} ^*\det \left( {\begin{array}{*{20}c} {q_{PA} } & {q_{PB} } \\ {q_{RA} } & {q_{RB} } \\ \end{array} } \right)\det \left( {\begin{array}{*{20}c} {\frac{{\partial f_{A} }}{\partial P}} & {\frac{{\partial f_{A} }}{\partial R}} \\ {\frac{{\partial f_{B} }}{\partial P}} & {\frac{{\partial f_{B} }}{\partial R}} \\ \end{array} } \right)$$

If the two determinants on the right hand side have opposite sign, the determinant of the Jacobi matrix is negative. In that case at least one of the eigenvalues is positive, and we have a saddle point. If the determinant is positive we can show that again the characteristic polynomial *λ*^4^ + *c*_1_*λ*^3^ + *c*_2_*λ*^2^ + *c*_3_*λ* + *c*_4_, with *c*_1_ minus the trace and *c*_4_ the determinant has all coefficients positive. That implies that if all eigenvalues are real, they must be negative. To rule out a complex pair with a positive real part, the Routh-Hurwitz criterion *c*_1_*c*_2_*c*_3_ > *c*_3_^2^ + *c*_1_^2^*c*_4_ must be satisfied. We have tested the criterion for a wide range of possible parameter values, and found it satisfied, but unfortunately we haven’t been able to show that to be the case generically. Note that the graphical representation in Fig. 1 does explain the stability. For slightly different cases there are proofs or other claims that indeed the coexistence point is stable (Léon and Tumpson 1975; Taylor and Williams 1975; Hsu et al. 1981; Butler and Wolkowicz 1987; Li and Smith 2001; Wu and Wolkowicz 2001). The only generic statement we can make is that if there is more than one coexistence point, these cannot all be stable. Since the *q*’s are fixed and consecutive coexistence points along the contour lines must have opposite signs for the determinant of the gradient matrix, no two consecutive points can be stable.
